# Skull Base Rhabdomyosarcoma Mimicking Osteomyelitis in a Pediatric Patient

**DOI:** 10.1055/a-2544-3543

**Published:** 2025-03-20

**Authors:** Avraham Adelman, Landon Richardson, Nikita Chapurin, Brian C. Lobo, Si Chen

**Affiliations:** 1 Division of Rhinology and Skull Base Surgery, Department of Otolaryngology–Head and Neck Surgery, University of Florida, Gainesville, Florida, United States; 2 Division of Otology/Neurotology, Department of Otolaryngology–Head and Neck Surgery, University of Florida, Gainesville, Florida, United States

**Keywords:** rhabdomyosarcoma, petrous apex, skull base tumor, skull base osteomyelitis

## Abstract

Rhabdomyosarcoma (RMS) is a rare malignant tumor, affecting 4.58 per 1 million children, with approximately 35% occurring in the head and neck. Skull base RMS commonly presents at advanced stages and delays diagnosis due to its overlapping features with other skull base pathology, and difficulty accessing the lesion for biopsy. This case illustrates these challenges in skull base RMS mimicking osteomyelitis of the petrous apex.

Case: A 6-year-old immunocompetent female, with a history of two acute otitis media episodes, presented with a 3-week history of sixth cranial nerve palsy and sudden-onset complete seventh cranial nerve palsy. She did not have pain or otorrhea. Computed tomography (CT) and magnetic resonance imaging revealed a 1.3 cm left petrous apex enhancing lesion with extension into the mastoid and clivus with surrounding bony and soft tissue destruction. A nuclear medicine scan (Technetium-99m followed by gallium) demonstrated avid uptake in the left petrous apex. The working diagnosis was skull base osteomyelitis, for which the patient received 2.5 weeks of antibiotics. After failing to improve, repeat imaging showed significant progression of the disease and extension into the nasopharynx and sphenoid sinus. An endoscopic trans-sphenoidal biopsy was performed with pathology consistent with RMS. CT chest revealed lung metastases. The patient partially responded to chemotherapy with vincristine, actinomycin-D, and cyclophosphamide alternating with vincristine and irinotecan. During week 13 of chemotherapy, she received concomitant proton therapy to a total dose of 5040 cGyRBE. Five months after diagnosis, she developed leptomeningeal spread, which was further complicated by meningitis, and passed away.

## Introduction

Rhabdomyosarcoma (RMS) is a malignant soft tissue tumor derived from embryonic mesenchymal cells. 1 With approximately 350 new cases per year in the United States, RMS is the most common soft tissue sarcoma in children, accounting for approximately 3% of all pediatric tumors. 2 3 The initial presentation of pediatric RMS varies widely based on the anatomical location, subtype, and disease stage. Approximately 35% of these emerge in the head and neck, with a proclivity for the orbit and para meningeal tissue. 4 5 Only 8 to 10% of head and neck RMS involve the temporal bone. 6 Symptoms such as painless swelling, rhinorrhea, sinusitis, otitis media, and cranial nerve deficits often mimic benign otolaryngological conditions, increasing the likelihood of delayed or missed diagnoses. This is particularly troubling because these delays may exacerbate the already poor prognosis associated with head and neck RMS, which often presents at an advanced stage. 7

Herein, we present a very rare case that underscores the challenges of diagnosing skull base RMS, given its overlap in presentation with osteomyelitis and difficulty in surgical access.

## Case Presentation

A 6-year-old immunocompetent girl with a 3-week history of left sixth cranial nerve palsy presented to the emergency department with new-onset facial weakness without pain or otorrhea. History revealed two recent episodes of acute otitis media treated with amoxicillin. The examination was positive for complete left seventh and sixth cranial nerve palsies. Leukocyte count was within normal limits, but both erythrocyte sedimentation rate and c-reactive protein were elevated.

**Fig. 1:**
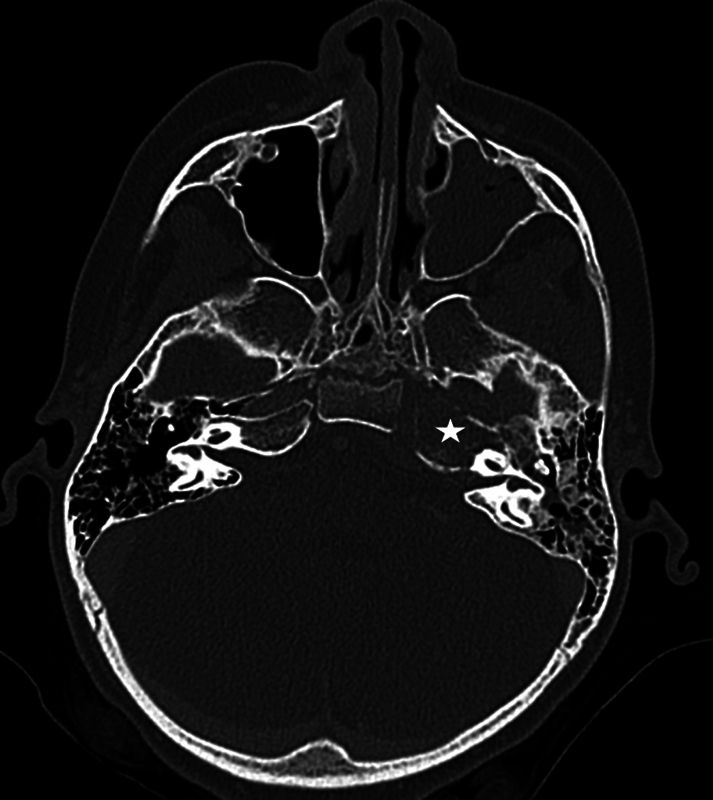
CT temporal bone demonstrating expansile lesion with bony destruction at the left petrous apex (star). There was left maxillary opacification and partial mastoid effusion.

**Fig. 2:**
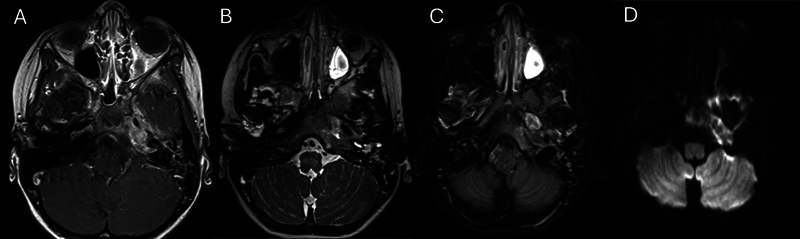
Left petrous apex on ( **A** ) T1 with contrast showed enhancement and inflammatory changes extending into the Meckel cave with dural thickening; ( **B** ) T2 with hyperintensity; ( **C** ) T2 fat sat demonstrated hyperintensity; ( **D** ) DWI restriction.

**Fig. 3:**
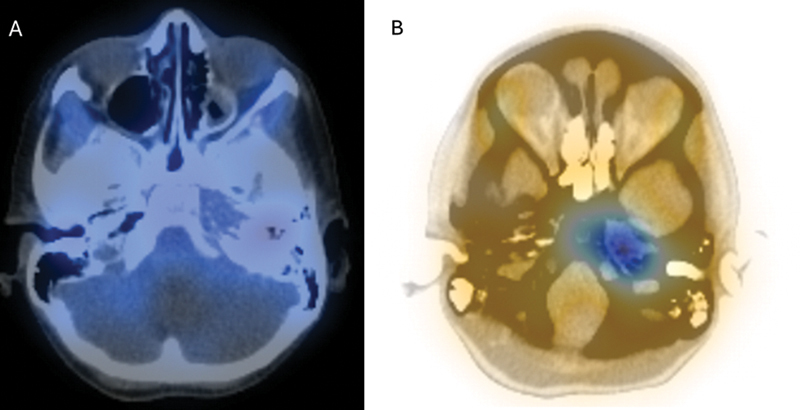
SPECT-CT ( **A** ) with technetium showed trace uptake ( **B** ) with gallium demonstrated extensive uptake in the left petrous bone, consistent with extremely active infection.

Computed tomography (CT) on admission revealed mucosal thickening of the left sphenoid sinus and mastoid, destructive changes centered on the left petrous apex, and phlegmonous changes from the petrous apex extending to the clivus ( Fig. 1 ). MRI revealed a 1.3 cm × 0.8 cm × 1.2 cm rim-enhancing lesion and destructive changes extending to the clivus ( Fig. 2 ). Nuclear medicine scan, with technetium-99m followed by gallium, demonstrated avid uptake in the left petrous apex ( Fig. 3 ).

The working diagnosis was left otomastoiditis complicated by skull base osteomyelitis of the petrous apex. After careful consideration of risk versus benefit, a biopsy and culture were deferred due to its location in the petrous apex, which would require significant surgical exposure and potential harm to vital structures. At the initial presentation, there was no clear corridor to access the lesion via the transnasal or transmastoid approach. The middle fossa approach was considered, however, deemed aggressive for biopsy in this young patient. The patient was thus started on intravenous ceftriaxone, vancomycin, and metronidazole, with ceftriaxone later switched to cefepime for pseudomonal coverage.

**Fig. 4:**
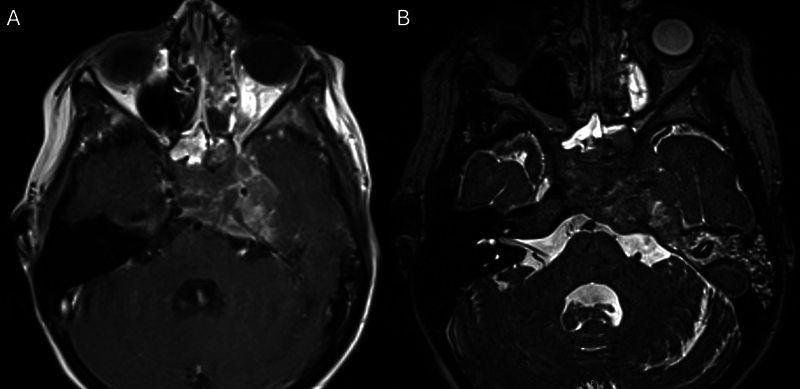
Repeat MRI at 2.5 weeks ( **A** ) T1 with contrast and ( **B** ) T2 demonstrated the rapid growth of the petrous apex lesion now extending into the nasopharynx and sphenoid sinus.

After 2.5 weeks of treatment, there was no improvement in cranial nerve function. Repeat MRI demonstrated significant expansion of the lesion, including into the nasopharynx and sphenoid sinus ( Fig. 4 ). This created a corridor for the endoscopic transnasal approach through which, a biopsy was obtained. Irregular, friable mucosa was sampled from the left fossa of Rosenmüller and the sphenoid sinus.

Pathology identified a FOXO1-negative embryonal RMS and cultures/gram-stain revealed no evidence of infection. Staging studies, including lumbar puncture and bone marrow biopsy, were negative for malignancy. However, a chest CT revealed bilateral lung metastases. The patient was diagnosed with Stage IV skull base RMS and started on a chemoradiation regimen. Chemotherapy agents utilized included vincristine, dactinomycin, and cyclophosphamide alternating with vincristine and irinotecan. After a partial response, starting at week 13 of chemotherapy, the patient underwent concomitant proton therapy to a total dose of 5040 cGyRBE. Five months after diagnosis, she developed leptomeningeal spread, which was further complicated by meningitis, and passed away.

## Discussion

RMS of the petrous apex is an exceedingly rare condition that can closely mimic infectious or inflammatory pathology such as osteomyelitis. The petrous apex of the temporal bone, a common site for skull base osteomyelitis as well as para meningeal RMS, poses significant surgical challenges due to its location embedded in the skull base and its vicinity to vital structures. Given its location, accessing the petrous apex poses a risk to the internal carotid artery, cranial nerves V to VIII, and other surrounding critical structures. 8 There are five main skull base approaches to reach the anterior petrous apex: endoscopic transnasal, translabyrinthine, transcochlear, transcranial infracochlear, and middle fossa craniotomy. 6 8 Lesions that are lateral to the vertical segment of the carotid are difficult to reach via the transnasal approach. This case was initially inaccessible except via transnasal and infracochlear approach, and the rest of the approaches were deemed aggressive for a 6-year-old patient for biopsy. Thus, empiric treatment of antibiotics was initiated. Alongside RMS, other common pathologies of the petrous apex include cholesterol granulomas, cholesteatomas, Langerhans Cell Histiocytosis, as well as skull base osteomyelitis, which can present similarly to RMS on imaging. 8

The patient received treatment for suspected skull base osteomyelitis, a condition that typically shows slow symptom resolution, resulting in widely varying follow-up imaging timelines. Monitoring treatment response tends to rely on clinical parameters, such as cranial nerve improvement, making it challenging to determine the optimal duration of treatment or the need for adjustments to the antibiotic regimen. 9 10 The role of repeat imaging during acute treatment is complex, as conventional modalities like CT and MRI tend to not accurately reflect disease activity. 10 While nuclear medicine modalities may provide better assessment of disease activity, it is overall common to not repeat imaging at all during the treatment course, unless there is clinical deterioration. 10 11 In the present case, biopsy and culture were not obtained due to lack of access to biopsy the lesion. Short-interval imaging revealed growth of the lesion into the nasopharynx and sphenoid sinus. Given the lack of response to antibiotics and the now significantly lower-risk location for access, the decision was made to biopsy via an endonasal approach.

The overall 5-year survival for pediatric RMS is 69%. 12 However, these para meningeal cases account for a disproportionately poor prognosis due to their commonly advanced stage upon clinical presentation and precarious location within the skull base, highlighting the need for diagnostic precision. 13

Multimodal imaging is key in diagnosing skull base RMS. MRI is particularly valuable in providing detailed insight regarding tumor origin, local involvement, and metastasis. 14 RMS typically appears as isointense on T1-weighted images with strong postcontrast enhancement and hyperintense on T2-weighted images. Restricted diffusion on diffusion-weighted imaging aids in additional tumor characterization and staging. 13 14 On CT, RMS appears isodense or slightly hypodense on precontrast scans with homogenous enhancement postcontrast. 14

Nuclear medicine scans are widely used for RMS evaluation but can be complicated by increased uptake resembling osteomyelitis on PET/CT, especially after gallium, as demonstrated in this case. 15 This problem extends to other imaging modalities as well: MRI is highly sensitive to detect acute osteomyelitis, but typical findings of T1 clival hypointensity and T2 hyperintensity closely resemble RMS. 15 CT findings are nonspecific for osteomyelitis and are usually limited to bone erosions and surrounding soft tissue swelling. 15 Nonspecific imaging findings can pose significant challenges in evaluating petrous apex pathology since biopsies cannot always be easily obtained. Positioned deep within the skull base, the petrous apex is challenging to access surgically due to the proximity of critical structures including the internal carotid artery, cavernous sinus, and Meckel's cave. 8

Due to their similarities, there have been reports of skull base osteomyelitis mimicking malignancy. Gomez et al described a 6-year-old girl presenting with diffuse headache, upper and lower extremity motor weakness, and 2 days of postprandial vomiting. MRI revealed a destructive central skull base lesion initially suspected to be a chordoma. However, attempted resection uncovered inflammatory tissue with purulent drainage, and a biopsy demonstrated *Streptococcus aureus* osteomyelitis. 16 In a similar scenario, Seki et al reported a 3-year-old girl with a 1-week history of vomiting, fever, and headache. Imaging showed a lesion centered on the clivus without bony destruction, which was suspicious of malignancy. However, while on empiric antibiotics, follow-up imaging revealed a decrease in the size of the mass, and *Streptococcus milleri* was identified in blood cultures, leading to the diagnosis of skull base osteomyelitis versus malignancy. 17

To our knowledge, this is the first case of a pediatric petrous apex RMS mimicking osteomyelitis. We illustrate the difficulties of diagnosing skull base RMS due to its overlap with other diagnoses and its location for surgical access. We hope to caution clinicians to consider skull base RMS in their differential diagnosis of suspected skull base osteomyelitis in children, prompting short-interval repeat imaging, especially when culture/biopsy cannot be obtained.
